# Exploring the networking behaviors of hospital organizations

**DOI:** 10.1186/s12913-018-3144-4

**Published:** 2018-05-08

**Authors:** Fausto Di Vincenzo

**Affiliations:** Department of Economic Studies, G. d’Annunzio University, Viale Pindaro 42, 65127 Pescara, Italy

**Keywords:** Networking behavior, Hospital organizations, Patient transfer, Social network analysis

## Abstract

**Background:**

Despite an extensive body of knowledge exists on network outcomes and on how hospital network structures may contribute to the creation of outcomes at different levels of analysis, less attention has been paid to understanding how and why hospital organizational networks evolve and change. The aim of this paper is to study the dynamics of networking behaviors of hospital organizations.

**Methods:**

Stochastic actor-based model for network dynamics was used to quantitatively examine data covering six-years of patient transfer relations among 35 hospital organizations. Specifically, the study investigated about determinants of patient transfer evolution modeling partner selection choice as a combination of multiple organizational attributes and endogenous network-based processes.

**Results:**

The results indicate that having overlapping specialties and treating patients with the same case-mix decrease the likelihood of observing network ties between hospitals. Also, results revealed as geographical proximity and membership of the same LHA have a positive impact on the networking behavior of hospitals organizations, there is a propensity in the network to choose larger hospitals as partners, and to transfer patients between hospitals facing similar levels of operational uncertainty.

**Conclusions:**

Organizational attributes (overlapping specialties and case-mix), institutional factors (LHA), and geographical proximity matter in the formation and shaping of hospital networks over time. Managers can benefit from the use of these findings by clearly identifying the role and strategic positioning of their hospital with respect to the entire network. Social network analysis can yield novel information and also aid policy makers in the formation of interventions, encouraging alliances among providers as well as planning health system restructuring.

## Background

Interest in understanding how and why hospital organizations choose collaborative partners overtime is a relatively recent issue and is related to a new strand of research that investigates these phenomena using concepts and methods from organizational sociology and network theory [1–5]. Networking behavior of organizations matters because they can achieve better performances, mitigate competition, learn by interaction, and develop effective ways to absorb external knowledge produced by their partners [6–9]. Networking matters also because organizations are connected to their environments through other organizations [10]. As a consequence, the quality, quantity and value of resources that an organization can access and the terms of availability of such heavily depend on the relations that it is able to establish with exchange partners [11].

Previous literature assumes that existing and past relations among organizations may act endogenously to induce networking [7, 12], and how the position that an organization occupies in the web of industry relations affects the formation of networking relationships [13]. Despite an extensive body of knowledge exists on network outcomes and on how network structures may contribute to the creation of outcomes, less attention has been paid to understanding how and why organizational networks emerge, evolve, and change [14].

A quantitative exploration based on social network analysis (SNA) and specifically on stochastic actor-based model for network dynamics [15–17] was employed to understand networking behavior dynamics of hospital organizations, and specifically to identify the endogenous and exogenous determinants underlying the propensity of hospitals to exchange network ties. The research relies on original fieldwork and longitudinal data on patient transfer relations within a regional community of hospital organizations in Italy. Patient transfer flows reflect collaboration and the existence of underlying relationships between the hospitals involved [1, 2]. Patient transfers between hospitals are directly observable and require high levels of coordination and communication [3]. The transfer of a patient requires the exchange of detailed clinical information which by definition, is complex due to the growth and specialization of clinical knowledge and the multiple combinations of conditions that patients can be subject to, and involves the co-construction of an understanding of the patient that needs also to consider the cognitive aspects of the actors involved in the exchange [18].

Recently, a number of studies have addressed the issues of the determinants of patient transfer between hospital organizations. In order to reduce staff uncertainty and coordinate their efforts, hospitals tend to routinize destination selection such that staff immediately contacted a “usual” transfer destination [19]. Transfer destination selection, therefore, was primarily driven at an institutional level by organizational concerns and bed supply, rather than physician choice or patient preference [19]. Remaining within the ambit of the organizational features, further studies have shown how patients are more likely to be transferred between hospitals differing in size [20], high-volume and larger hospitals are more attractive partners than small hospitals based on their greater availability of resources and infrastructures [21], resource complementarity especially in terms of technological assets and expertise matter in explaining the propensity of hospital to collaborate [22], and that patients often move from low-performance hospitals to high-performing hospitals [1, 20]. Among the institutional variables, it was highlighted how patients are more likely to be transferred between hospital belonging to the same Local Health Authority (LHA) and having the same organizational forms (ownership-governance structure) [3]. Finally, the literature analyzed the impact of the geographical variable, highlighting how geographically proximate hospitals were somewhat more likely to share patients [2, 23].

Despite this abundance of studies, most of them have in common the limit of being studies with a cross-sectional data setting or that have not been pushed to longitudinally analyze the evolution of patient transfer dynamics in a wide span of time. There are two researches that, however, are an exception to this limitation. The first, conducted by Lomi et al. [4], observed patient sharing events between hospitals during four consecutive years finding that quality of care, measured as 45-day risk-adjusted readmission rate, has an impact on the propensity of hospital organizations to exchange patients over time. The second, conducted by Stadtfeld et al. [5], explains assimilation and differentiation mechanisms (among which the propensity to transfer patients) between network partners over time. However, currently, no studies have already provided a longitudinal investigation of the determinants of patient transfer evolution employing stochastic actor-based model for network dynamics, and modeling partner selection choice as a combination of multiple organizational attributes and endogenous network-based processes. The present study aims to fill this gap in the literature.

## Methods

### Research setting

The dynamics of patient transfer relations within the entire network of hospitals providing services to patients in Abruzzo, a region in central Italy with a population of approximately 1,300,000 residents, have been analyzed. The Italian National Health Service (I-NHS) is a publicly funded health system that provides universal coverage. The government, at the central level, allocates resources to 20 Italian regions and is responsible for defining the core benefit packages and ensuring that basic coverage is provided to the entire population. Regional governments have wide autonomy in planning, allocating resources, and organizing regional level services, and are responsible for delivering health care services to their resident populations.

The Abruzzo regional health system is entrusted to six LHAs, and health care services are provided by 35 hospital organizations (22 public and 13 private). Of the 22 public hospitals, two are teaching hospitals. Public hospitals provide highly specialized hospital care and are characterized by technical, economic, and financial autonomy. Teaching hospitals are hospitals linked to universities, and provide education, research, and tertiary care. Private hospitals are partially financed by the regional healthcare service and are investor-owned organizations that provide ambulatory assistance, hospital care, and diagnostic services.

The study setting seems to be particularly appropriate for the purpose of this research. The first reason is that earlier research in this context [3, 24, 25] and the fieldwork show the presence of local networks of collaboration among hospitals, which mainly stem from the transfer of patients between hospitals. Patient transfer occurs when one hospital directly transfers one or more elective patients to another hospital. For example, hospitals that provide only basic services may send patients with more complicated clinical problems to another provider that offers comprehensive specialty care. Patient transfer may also be driven by ‘asymmetries’ in regional providers’ clinical resources or competences: e.g., hospitals may transfer patients to other local providers if they lack the necessary medical equipment (e.g., intensive care unit beds), expertise (e.g., staffing), or supplies. These informal networks become established and can have important implications for organizational performance [24, 25].

The second reason is that, given the great strategic and organizational autonomy of our empirical setting, there are no significant external factors that influence the networking process for which to control. In the period considered in this study, there were no significant policy interventions that substantially altered the institutional framework, the number of providers, or the structure of the local inter-organizational network. Exception is the progressive reduction in the number of beds set by regional authorities, but this has affected proportionally all hospitals.

The third reason why this is an ideal case to study network dynamics is that Abruzzo health care system suffers from a lack of systemic planning and strategy coordination among its hospitals [26]. Unlike some other regions that have fostered inter-hospital collaboration through well-defined and formal collaboration mechanisms (e.g., “hub&spoke” models or clinical pathways for patient referrals), coordination in Abruzzo emerges mainly through patient transfer among providers [3]. Especially in regions where systemic planning and organizing of health provision is lacking, collaborative initiatives among hospitals arise and evolve endogenously [26]. These emergent “self-organizing” properties of inter-hospital networks may produce outcomes and behaviors that can be investigated by employing longitudinal models and social network analysis [15].

### Data collection

The analysis draws on a range of rich data. Data on patterns of collaborative interdependencies during the period 01/01/2003–31/12/2008 among all hospitals in the region were extracted from the hospital information system database managed by the Abruzzo Region. Data on hospital activities, and information on demographics and performance, were taken from the Abruzzo Health Agency archives and yearly reports. These data are collected regularly and archived digitally by the Region for administrative purposes, and by the Health Agency for its operational and reporting activities. Archival sources are generally more precise and detailed than surveys and provided complete information on the network of hospitals: there were no missing data.

### Variables

#### Dependent variable

Since this study is interested in understanding the dynamics of networking behavior of hospital organizations, the dependent variable is inter-hospital collaboration measured as transfers of patients [1, 2, 22]. Using available data on patient transfer among regional hospitals, as dependent variable, six “35 × 35” dichotomized matrices one for each of the years from 2003 to 2008 were built. The rows and columns of each matrix respectively report the hospitals that sent and admitted at least one patient between January 1 and December 31 in each of the year considered. Because matrices may vary depending on the dichotomization criteria, separated analyses were conducted to assess the effect on the results of different criteria (i.e. “greater-than” mean value, “greater-than” zero). The results obtained were qualitatively similar.

#### Explanatory variables

This research tested for several organizational-level variables that might influence networking behavior of hospital organizations. Specifically:

##### Case mix

Measure of the level of complexity of the cases which are treated in a given hospital. It measures if hospitals facing with highly complex cases (for example, transplants, stroke, or hearth attacks) have different networking behavior compared to hospitals treating patients with a low degree of disease severity (for example, appendicitis, rehabilitation, etc.).

##### N° of common specialties

It counts the number of overlapping specialties, and indicates to what extent two hospitals are alike because they do the same thing or not. It serves to investigate whether the transfer of patients occurs between hospitals that overlap in knowledge stocks. Using available data on specialties (clinical wards) present in each hospital in the region, was built a “35 × 35” matrix. The rows and columns of the matrix respectively report the hospitals in the Region, while the cells of intersections report the number of overlapping specialties between each pair of hospitals. The matrix was computed for the year 2003 and was regarded as a constant in the statistical model as there have not been major changes in the number and types of specialties present at each hospital during the six years.

##### Staffed beds

A proxy of dimension, measured as the number of staffed beds [1, 20].

##### LHA membership

It considers the affiliation of hospitals to the distinct LHAs in which the region is divided [3]. In a “35 × 35” matrix, the rows and columns of the matrix respectively report the hospitals in the Region, while the cells of intersections report 1 if pairs of hospitals were affiliated to the same LHA, 0 otherwise.

##### Performance

Measured as productivity, has been computed as the total number of admissions adjusted for case mix, divided by total number of staffed beds [24].

##### Geographical distance

A “35 × 35” matrix, the rows and columns of the matrix respectively report the hospitals in the Region, while the cells of intersections report the distance between each pair of hospitals expressed in km [23].

##### Percentage of emergency admissions

It represents unplanned emergency admissions as a percentage on the total admitted patients, as in previous studies [27]. It is commonly used as proxy of the level of uncertainty of input (i.e. patients) faced by the hospital [22].

Each variable was computed yearly, for the six-year period 2003–2008. Table 1 presents the descriptive statistics for the independent variables used in this research.

**Table 1:** Descriptive statistics of independent variables

Variables	Changing/ Constant	2003				2004				2005				2006				2007				2008			
		Mean	StD	Min	Max	Mean	StD	Min	Max	Mean	StD	Min	Max	Mean	StD	Min	Max	Mean	StD	Min	Max	Mean	StD	Min	Max
LHA	*Constant*	3.00	1.81	1.00	6.00																				
Staffed beds	*Changing*	183.03	164.68	20.00	730.00	167.83	146.70	20.00	661.00	168.17	145.27	20.00	650.00	167.83	146.70	20.00	661.00	167.83	146.70	20.00	661.00	132.97	129.73	20.00	572.00
Case-mix	*Changing*	1.03	0.13	0.81	1.40	1.03	0.13	0.81	1.40	1.02	0.12	0.70	1.42	1.03	0.11	0.66	1.29	0.92	0.20	0.00	1.24	0.90	0.12	0.66	1.22
N° common specialties	*Constant*	15	7.32	1	31																				
Productivity	*Changing*	35.65	16.80	5.45	79.40	42.92	23.13	9.21	130.44	50.93	27.10	11.21	136.37	32.49	16.24	5.74	77.16	28.10	14.72	0.00	60.00	38.10	19.78	6.51	103.49
Emergency. admissions	*Changing*	31.657	25.53	0	71	35.74	24.25	0.00	71.00	37.35	24.54	0	74	34.77	28.07	0	79	34.69	27.44	0	75	31.26	25.55	0	71
Geographic distance	*Constant*	65.69	29.61	0	133																				

Geographical distance between hospitals, LHA membership and the number of specialties are constant over time, showing the absence of structural policies for the re-designing of the hospitals regional system. The mean of staffed beds reduces over time (such as the percentage of emergency admissions) while the case mix complexity and the productivity indicators slightly increase in the six years analyzed. Table 2 reports correlations among all the variables included in the full model.

**Table 2:** Correlations

		1	2	3	4	5	6	7	8	9	10	11	12	13	14	15	16	17	18	19	20	21
1	Outdegree	–																				
2	Reciprocity	0.159																				
3	Transitive ties	0.381	0.375																			
4	3-Cycles	− 0.087	−0.147	−0.597																		
5	Balance	−0.335	− 0.478	− 0.915	0.405																	
6	Indegree-popularity	−0.690	− 0.208	− 0.743	0.370	0.658																
7	Outdegree-activity	− 0.495	− 0.546	− 0.855	0.203	0.913	0.649															
8	LHA	− 0.185	−0.081	0.297	− 0.205	− 0.286	− 0.112	− 0.210														
9	N° common specialties	0.126	0.097	−0.033	0.017	0.021	− 0.018	0.013	− 0.075													
10	Geographical distance	−0.047	− 0.113	− 0.371	0.138	0.359	0.307	0.363	0.305	0.103												
11	Staffed beds (alter)	0.003	−0.166	−0.048	− 0.079	0.099	− 0.303	0.195	0.086	−0.218	− 0.008											
12	Staffed beds (ego)	0.358	0.346	0.448	0.049	−0.521	− 0.290	− 0.707	0.159	− 0.164	− 0.204	− 0.112										
13	Staffed beds (similarity)	−0.490	− 0.311	− 0.595	0.151	0.587	0.582	0.678	−0.123	−0.257	0.241	0.419	−0.275									
14	Case-mix (alter)	−0.018	− 0.130	− 0.181	0.068	0.183	0.152	0.181	−0.077	0.001	0.097	−0.098	− 0.055	0.181								
15	Case-mix (ego)	−0.079	− 0.018	0.007	−0.008	0.003	0.020	−0.009	0.041	0.011	−0.048	0.127	−0.042	0.095	−0.043							
16	Case-mix (similarity)	0.147	0.208	0.169	−0.044	−0.198	−0.147	− 0.204	−0.001	0.105	−0.026	− 0.060	0.087	− 0.218	−0.420	− 0.070						
17	Emergency adm. (alter)	0.044	−0.247	−0.023	− 0.176	0.119	− 0.189	0.156	0.009	−0.041	− 0.054	0.164	− 0.162	0.063	− 0.141	0.084	− 0.066					
18	Emergency adm. (ego)	−0.247	−0.066	− 0.187	0.150	0.204	0.181	0.096	0.061	−0.123	−0.040	− 0.016	−0.055	0.007	0.086	0.079	−0.144	0.118				
19	Emergency adm. (similarity)	−0.012	0.067	0.244	−0.210	−0.333	− 0.101	−0.225	0.115	−0.111	− 0.259	−0.071	0.117	−0.156	− 0.063	−0.075	0.022	−0.235	− 0.271			
20	Productivity (alter)	0.033	0.148	0.120	0.028	−0.182	−0.132	− 0.182	0.112	0.062	0.047	−0.248	0.077	−0.292	−0.104	− 0.162	0.001	− 0.040	−0.042	0.031		
21	Productivity (ego)	0.057	−0.081	−0.067	− 0.064	0.114	0.053	0.109	0.088	0.001	0.177	0.029	−0.244	0.046	−0.020	− 0.038	− 0.049	0.072	0.038	−0.058	− 0.200	
22	Productivity (similarity)	−0.096	0.118	0.208	−0.089	−0.200	− 0.043	− 0.201	0.095	−0.010	− 0.078	−0.170	0.083	−0.236	− 0.116	−0.087	− 0.147	−0.294	− 0.139	−0.032	0.482	−0.056

In the estimation, the model controlled also for some structural endogenous effects named respectively outdegree, reciprocity, transitive ties, three cycles, balance, indegree-popularity, and outdegree–activity. Table 3 describes in detail each of the types of relational patterns investigated and how they should be interpreted. Only these simple and basic effects and not for the more sophisticated ones have been included in the model because they represent the most commonly used in works that exploits stochastic actor-based models [28, 29].

**Table 3:**
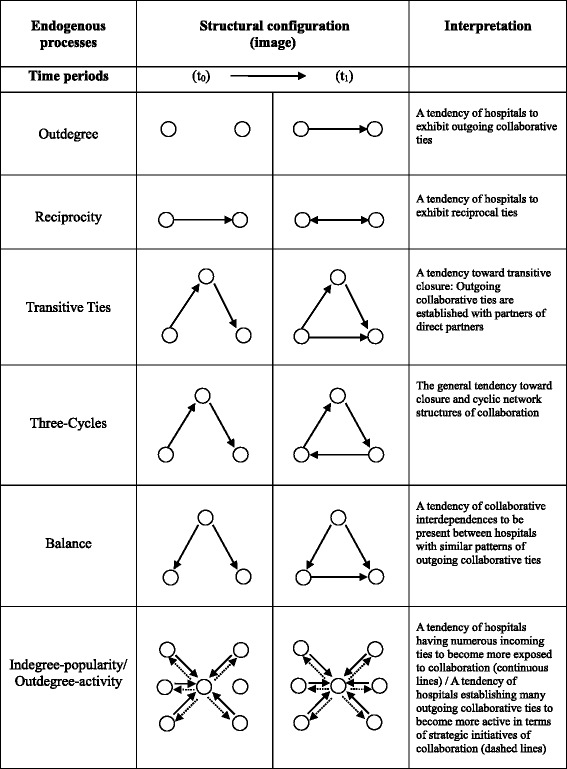
Summary of structural configurations

### Estimation technique

The R-Siena Software Package [15] allowed to conduct the exploratory analysis. The observed changes can be explained as functions of both individual and dyadic characteristics of actors and structural effects. Specific actor attributes and dyadic characteristics either favor or reduce the probability that two hospitals will transfer patients and so collaborate. For each actor and dyadic attribute, several effects have been included in the model specification. As explained by Snijders et al. [15], for continuous actor covariates (e.g., staffed beds, case mix, productivity, emergency admissions), three kinds of actor-driven mechanisms can be specified. The sender (ego) and receiver (alter) effects evaluate the tendency for organizations with higher attributive value to, respectively, send out more (higher outdegree) or receive more (higher indegree) than others. The “similarity” effect measures whether collaborative relations tend to occur more often between organizations with similar values for a given attribute. Finally, for the constant dyadic (LHA membership and overlapping specialties), the effects included in the model measure the tendency for ties between actors with the “same” value of that variable. Structural effects represent endogenous network mechanisms that also may influence the probability of interdependence between actors. For examples of introductory papers employing stochastic actor oriented models the reader can refer to Snijders, van de Bunt and Steglich [15]. For a more mathematical treatment and definition of effects in such models for network dynamics the reader can refer to Ripley et al. [29].

## Results

Table 4 reports key statistics describing the evolution of network ties in terms of density (i.e. ratio of number of collaborative ties observed yearly on the total number of possible ties), average degree (i.e. average number of collaborative partners for each node), and total number of ties. With the exception of the year 2006, density and number of ties increased slightly from 11% in 2003 to 12.2% in 2008, and from 131 in 2003 to 145 in 2008 respectively. Also, in the six-year period observed, the average number of collaborative ties increased from 3.743 to 4.143.

**Table 4:** Characteristics of ties evolution

Year	Density (%)	Number of ties	Average degree
2003	11	131	3.743
2004	10.2	121	3.457
2005	11.2	133	3.800
2006	9.5	113	3.229
2007	11.5	137	3.914
2008	12.2	145	4.143

To explore networking behavior dynamics more in-depth, have been also considered the collaborative patterns at dyadic level over time (see Table 5). The column labeled 0 → 0 reports the number of pairs of hospitals that did not develop a collaborative relationship in the observed wave; the column labeled 1 → 1 indicates the number of pairs that maintained their collaborative relationships. The other two columns report the number of ties formed or dissolved from one year to the next. Consistent with the third column in Table 4, also Table 5 shows a growing trend in tie changes: during the period of observation, 213 new collaborative ties were formed and 199 existing relationships were dissolved. The Jaccard coefficient is a measure of similarity for two sets of data, with a range from 0% to 100%. The higher is the percentage, the more similar are the two networks of hospitals. The values in Table 5 show that around 50% of ties, over time, change between subsequent observations from one year to another.

**Table 5:** Number of changes between subsequent observations

Observation time	0 → 0	0 → 1	1 → 0	1 → 1	Jaccard
2003–2004	1030	29	39	92	0.575
2004–2005	1022	47	35	86	0.512
2005–2006	1029	28	48	85	0.528
2006–2007	1021	56	32	81	0.479
2007–2008	1000	53	45	92	0.484

Columns labeled “0➔1” and “1➔0” indicate the number of forming and dissolving collaborative ties, respectively, from one period to the next. Columns labeled “0➔0” and “1➔1” indicate the number of pairs for which no changes were observed

The empirical results of the stochastic actor based model estimations are presented in Table 6. The analysis of the endogenous effects suggests that collaborative ties do not evolve randomly but instead follow specific relational patterns. The significant negative effect of outdegree and the significant positive effect of reciprocity respectively indicate a general tendency of organizations against outgoing collaborative ties and the propensity overtime to reciprocate received collaborative ties. The significance of these two basic measures is crucial in stochastic actor-based model for network dynamics as they provide robustness to the entire model [15]. A lack of their significance would imply that the phenomenon object of investigation (i.e. the propensity to exhibit collaborative ties) is not statistically relevant. In addition, the network presents overtime a general tendency toward transitive ties, meaning as collaborative ties tend to be established with partners of direct partners. This is in line with the study results of Madhavan, Gnyawali and He [30] on triads formation in cooperative networks. The remaining endogenous effects are not statistically significant.

**Table 6:** Parameter estimates predicting the dynamics of collaborative interdependences

	Estimate	Standard Error	*p-value*	
Independent variables				
N° common specialties	−0.405	0.147	0.048	*
Case-mix (alter)	−0.891	0.413	0.584	
Case-mix (ego)	0.866	0.452	0.612	
Case-mix (similarity)	−0.921	0.647	0.039	*
LHA	1.330	0.154	< 0.000	**
Staffed beds (alter)	0.002	0.001	0.002	**
Staffed beds (ego)	0.001	0.001	0.163	
Staffed beds (similarity)	−0.228	0.546	0.376	
Geographical distance	−0.013	0.003	< 0.000	**
Productivity (alter)	0.004	0.004	0.842	
Productivity (ego)	−0.003	0.004	0.772	
Productivity (similarity)	1.704	0.699	0.061	
Emergency admissions (alter)	0.005	0.003	0.824	
Emergency admissions (ego)	0.018	0.003	0.072	
Emergency admissions (similarity)	0.902	0.256	0.050	*
Endogenous processes				
Outdegree	−2.576	0.177	< 0.000	**
Reciprocity	0.717	0.205	< 0.000	**
Transitive ties	0.771	0.234	0.006	**
Three-cycles	−0.164	0.097	0.583	
Balance	0.072	0.052	0.251	
Indegree-popularity	0.029	0.027	0.121	
Outdegree-activity	−0.126	0.074	0.341	

*p < 0.05; **p < 0.01. *P*-values are based on approximately normal distributions of *t-*ratios (defined as the parameter estimate divided by the standard error)

The coefficient of N° of common specialties is negative and significant. This implies that there is a negative relationship between the similarity in terms of overlapping specialties and the propensity of organizations to collaborate. Higher levels of overlapping specialties have a negative impact on networking behavior of hospital organizations, reducing their propensity to exchange collaborative ties.

The variable case-mix (similarity) is negatively and significantly correlated with the dependent variable, meaning that network ties are more likely to be observed among organizations with different values in this index. Geographical distance is negative and significant. This implies that there is a geographical proximity effect [2], namely that as the distance decreases the propensity of hospital organizations to collaborate increases.

In line with previous studies [22, 27], the results show that input uncertainty matter in explaining how hospital organizations choose their collaborative patterns. The variable Emergency admissions (similarity) is positive and significant, revealing a propensity to transfer patients between hospitals facing similar levels of operational uncertainty.

With reference to the proxy of size, staffed beds (alter) is positive and significant, showing a tendency in the network to choose larger hospitals as partners to which transfer patients [20].

The proxy used to measure whether similarities or differentials in performance levels (ie productivity) induce the networking behavior of hospitals organizations is not significant. It follows that if on the one hand it is widely recognized in literature that higher levels of collaboration produce a positive impact on organizational performance [24], on the other hand - at least in this specific case study - the opposite relationship is not true and therefore performance doesn’t matters in explaining partner selection.

Finally, the variable LHA is positive and significant, meaning that collaborative ties are more likely to develop between hospital organizations belonging to the same LHA.

## Discussion

Understanding the factors that stimulate or hinder networking behavior of organizations is a matter of significant theoretical interest and has remained high on the list of priorities of researchers interested in network relations [14]. Using stochastic actor-based model for network dynamics [15–17, 29], the purpose of this paper was to model partner selection choice as a combination of individual organizational attributes and endogenous network-based processes. The opportunity was provided by the availability of very rich data and the identification of an ideal empirical context within a regional community of hospital organizations in Italy.

The networking behavior of hospital organizations was observed through the study of patient flows, since the previous literature has amply demonstrated to us how this represents a valid proxy reflecting collaboration and the existence of underlying relationships between the hospitals involved in patient transfers, because of the high levels of coordination and communication that patient transfer requires [1, 2, 22, 25]. Although recently numerous studies have addressed the issue of patient transfer and investigated its determinants, this research topic was still uncovered as regards the study of evolutionary dynamics and factors that over time can induce or prevent ties formation among hospital organizations.

This research found that high levels of overlapping specialties reduce the propensity to exchange collaborative ties. This seems to suggest that perceiving another hospital organization as similar (in terms of dependence on the same resources, i.e. inputs represented by the patients), in this context seems to increase the competition between similar organizations and consequently inhibits the formation of network ties. Indeed, when two hospitals have many overlapping specialties, it implies that they are vey similar, they take care of the same diseases and treat the same type of patients, and thus may perceive each other as potential competitors [22]. Also this research found that, over time, network ties are more likely to be observed among hospital organizations that face with different case-mix. This seems to suggest that networking behavior is driven by clinical knowledge stock owned by a given hospital. Hospitals can suffer for the lack of high specialized physicians and nurses skilled to work in the intensive care unit, coronary care unit, stroke unit or operating surgery rooms equipped for transplantation, so they send patients to hospitals which could offer appropriate care related to patients’ pathologies [18]. In addition to the case-mix, the analysis also reveals a tendency in the network to choose larger hospitals as partners to which transfer patients, probably due to the fact that larger hospitals are more equipped in terms of resources and technologies [1, 20].

The results of the empirical analysis show that, over time, collaborative ties are more likely to develop between hospital organizations belonging to the same LHA. This can be interpreted in line with what was found by Veinot et al. [19], i.e. patient transfer is not considered by hospitals only as a solution to contingent one-off problems, rather it happens in a structured and localized social context where hospital organizations tend to routinize destination selection in order to coordinate their efforts and conserve their cognitive resources for patient care. The significance of the geographical dimension shows how the search for partners is also guided by proximity. This result, therefore, confirms what found by Mascia and colleagues [23] and extends the validity of their findings longitudinally to a wider time frame.

The last explanatory variable is operational uncertainty, that manifests when internal organizational activities are difficult to plan, or planned activities are difficult to execute, and it comes from unpredictable variation in internal operating conditions, which require change in original plans and routines, and the revision of resource allocation decisions. Although it is well known in the literature that health care organizations respond to uncertainty by creating ties [22, 27], this study adds that within the network there is a propensity over time to choose as collaborative partner hospitals facing similar levels of operational uncertainty. Future studies will have to clarify the motivations and theoretical mechanisms that explain this criterion in the choice of the partner organization.

Finally, results found that the formation of network ties between organizations is explained by peculiar forms of structural (or local) configurations, composed of subsets of two or three network actors and the possible ties among them [30]. These dyadic and triadic micro-processes have been measured statistically to provide evidence on how endogenous local forces drive the formation (and evolution) of network ties. Among the dyadic configurations, outdegree (the overall tendency of organizations to exhibit outgoing collaborative ties) and reciprocity (the overall tendency of organizations to exhibit reciprocal ties) are significant and confirm the non-static nature of the network investigated. Among the triadic configurations, only transitive ties (the tendency toward transitive closure, where collaborative ties are established with partners of partners) are significant in explaining how networks ties evolve over time [22, 28].

To reduce the risk of over-interpreting the results, it is useful to reflect on the main limitations of this research, which provide opportunities for future research. First, one specific relation, i.e. patient transfer among hospitals, was analyzed. Although inter-hospital collaboration is widely used in the literature [3], it is possible that hospitals also collaborate in other ways including exchanges of doctors, cross training of medical staff, and technology transfer. Future studies should pay attention to the multiplexity that inter-organizational collaboration is likely to involve. Second, our findings are based on data for a six-year period from hospitals in a single region of Italy and may reflect issues specific to the local context or the time period. Further research is encouraged on the dynamics of collaboration, in order to extend the application of longitudinal models for social network analyses to other settings, and to check whether our findings can be generalized.

## Conclusions

This study provides new insights by addressing the application of longitudinal models for social network analysis, which so far have received scant attention in health care. Delivery of hospital services is highly influenced by the formation of collaborative networks between providers. Hospital managers and policymakers are invited to use network analytic techniques that allow them to be informed about the current collaborative network. Also, through the use of these tools they can obtain novel information and understand better the effects of these networks, supporting the formation of structured agreements between hospitals, and allowing to draw proper patient flow at the regional level.

Health care networks are strongly self-organizing and emergent in nature, independent from (or even negatively influenced by) management and policymakers’ interventions (absence of interventions). It is therefore recommended to the latters of carefully define organizational characteristics (such as number of specialties, case-mix, size), institutional factors (LHAs) and geographical proximity as they count in determining the formation and shaping over time of hospital networks.

## Abbreviations

I-NHS: Italian National Health Service
LHA: Local health authority
